# Suspected Scrotal Metastasis Detected by ^18^F-FDG PET/CT in Recurrent Urachal Adenocarcinoma Associated with Subsequent High-Grade Urothelial Carcinoma: A Case Report

**DOI:** 10.1159/cro/abzag038

**Published:** 2026-08-27

**Authors:** Fatemeh Saboktakin, Mohammad Reza Ghasri, Shaghayegh Nourbakhsh, Najmeh Ranjbar, Sarvenaz Mirzakhani

**Affiliations:** Department of Nuclear Medicine, Imam Khomeini Hospital Complex, Tehran University of Medical Sciences, Tehran, Iran

**Keywords:** Urachal adenocarcinoma, Urachus, Scrotal metastasis, Urinary bladder, Urothelial carcinoma

## Abstract

**Introduction:**

Urachal adenocarcinoma is a rare and aggressive malignancy arising from persistent urachal remnants and accounts for <1% of bladder cancers. Although the lungs, liver, lymph nodes, bone, and peritoneum are recognized sites of metastatic spread, involvement of scrotal structures is exceedingly uncommon. We report a case of recurrent urachal adenocarcinoma with an unusual pattern of metastatic dissemination identified on ^18^F-FDG PET/CT.

**Case Presentation:**

A 59-year-old man underwent partial cystectomy with en bloc urachal resection and bilateral pelvic lymph node dissection for a moderately differentiated urachal adenocarcinoma arising from a persistent urachal remnant. Histopathology demonstrated a 6-cm tumor with perivesical extension and perineural invasion, while surgical margins and lymph nodes were negative. Approximately 6 months later, local recurrence was detected, and transurethral resection revealed high-grade urothelial carcinoma with supportive immunohistochemical findings. Restaging ^18^F-FDG PET/CT demonstrated a hypermetabolic recurrent bladder mass accompanied by pulmonary nodules suspicious for metastatic involvement, multiple intraperitoneal metastatic deposits, and an FDG-avid lesion within the right hemiscrotum highly suspicious for metastatic involvement.

**Conclusion:**

This case highlights the aggressive behavior and heterogeneous clinical course of urachal malignancies. In addition to confirming local recurrence, ^18^F-FDG PET/CT identified a lesion within the right hemiscrotum that was highly suspicious for metastatic involvement. Recognition of atypical metastatic patterns may improve disease staging and influence clinical management in patients with recurrent urachal carcinoma.

**Key Clinical Message**

Urachal adenocarcinoma may recur despite apparently complete surgical resection and can present with atypical metastatic dissemination. In patients with suspected recurrence, ^18^F-FDG PET/CT may reveal unexpected metastatic sites, including rare scrotal involvement, and provide comprehensive assessment of disease extent for treatment planning.

## Introduction

The urachus is an embryologic tubular structure derived from the involution of the allantois and cloaca during fetal development. Under normal circumstances, it regresses after birth and persists as the median umbilical ligament. Incomplete obliteration may result in persistent urachal remnants, including patent urachus, urachal sinus, urachal diverticulum, or urachal cysts. Although these anomalies are generally benign, malignant transformation can occur in rare circumstances, giving rise to urachal carcinoma [1, 2].

Urachal carcinoma is an uncommon but highly aggressive malignancy, accounting for <1% of all bladder cancers and approximately 0.01% of adult malignancies [1, 3]. Adenocarcinoma represents the predominant histologic subtype and constitutes >80–90% of urachal malignancies [1, 4]. Owing to its extraperitoneal location and frequently indolent clinical course, the disease often remains clinically silent until it reaches an advanced stage. Consequently, many patients present with locally invasive tumors or metastatic disease at diagnosis, contributing to the relatively poor long-term outcomes associated with this neoplasm [1, 3, 5].

Because of its rarity, current knowledge regarding urachal adenocarcinoma is largely derived from retrospective institutional experiences and case reports rather than prospective clinical trials [3, 6]. Complete surgical excision remains the cornerstone of treatment for localized disease and typically consists of partial cystectomy with en bloc resection of the urachus and umbilicus, often combined with pelvic lymph node dissection [1, 6]. Nevertheless, recurrence remains a major clinical challenge even after apparently complete resection. Several adverse prognostic factors have been associated with inferior outcomes, including positive surgical margins, lymph node involvement, peritoneal extension, advanced stage, and metastatic disease at presentation [1, 5, 6]. Despite advances in surgical management, recurrence rates remain substantial, and long-term surveillance is therefore essential [3, 5].

The metastatic behavior of urachal adenocarcinoma is heterogeneous and incompletely understood. The most frequently reported metastatic sites include the lungs, liver, lymph nodes, bone, and peritoneum [3, 5, 7]. However, unusual patterns of dissemination have occasionally been described, including metastases to anatomically unexpected locations. Such atypical presentations are of particular interest because they may provide insight into the biological behavior and metastatic pathways of this rare malignancy. Furthermore, the recognition of uncommon metastatic sites may have important implications for staging, treatment planning, and prognosis.

Accurate assessment of disease extent is therefore critical in patients with recurrent or metastatic urachal carcinoma. Although contrast-enhanced computed tomography (CT) remains the primary imaging modality for diagnosis and staging, increasing evidence supports a complementary role for ^18^F-fluorodeoxyglucose (^18^F-FDG) PET/CT. Recent studies have demonstrated that FDG PET/CT can identify metastatic lesions not appreciated on conventional anatomic imaging, resulting in disease upstaging and modification of therapeutic management in a substantial proportion of patients [7, 8]. Moreover, PET/CT may provide valuable whole-body evaluation in patients with suspected recurrence, particularly when unusual metastatic dissemination is present.

Herein, we report a highly unusual case of recurrent urachal adenocarcinoma arising from a persistent urachal remnant in a 59-year-old man who developed extensive metastatic disease despite margin-negative surgical resection and node-negative pathology. Restaging ^18^F-FDG PET/CT demonstrated local recurrence accompanied by pulmonary, peritoneal, and scrotal metastatic involvement. In addition, histopathologic evaluation of the recurrent lesion revealed high-grade urothelial carcinoma with supportive immunohistochemical findings. To our knowledge, scrotal involvement in the setting of recurrent urachal adenocarcinoma is exceptionally rare. Given the unusual metastatic distribution and the pivotal role of FDG PET/CT in defining the full extent of disease, we present this case together with a focused review of the available literature.

## Case Presentation

A 59-year-old man was referred to our institution for restaging with ^18^F-FDG PET/CT following histopathologic confirmation of recurrent bladder malignancy. The patient had a history of a persistent urachal remnant associated with a mass arising from the bladder dome. Initial contrast-enhanced CT demonstrated a urachal cystic lesion with focal mural thickening and a mass-like component measuring approximately 34 mm at the urachal-bladder junction, raising suspicion for urachal malignancy (Fig. 1).

**Fig. 1.**
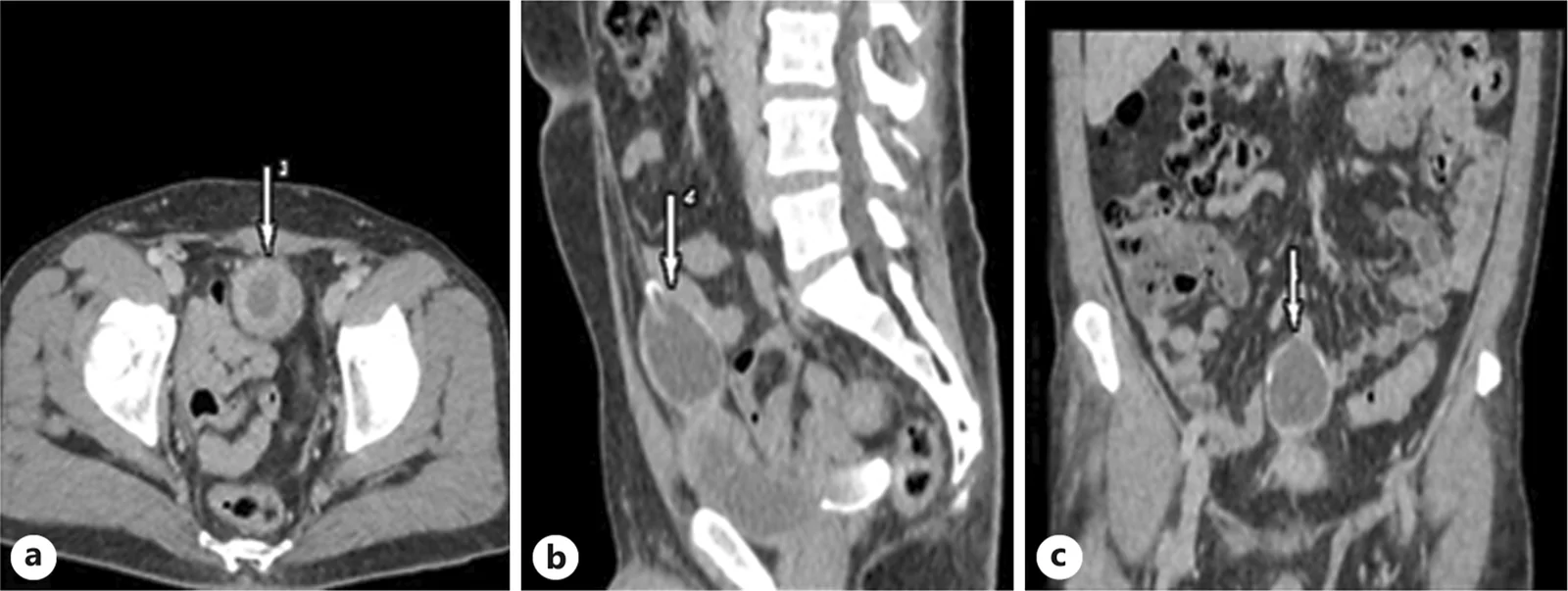
Preoperative contrast-enhanced CT findings of urachal adenocarcinoma. **a** Axial contrast-enhanced CT demonstrating a mixed cystic-solid lesion arising from the urachal remnant at the urachal-bladder junction (arrow). **b** Sagittal reformatted image showing the midline urachal mass extending from the bladder dome toward the anterior abdominal wall (arrow). **c** Coronal image demonstrating the craniocaudal extent of the lesion along the expected course of the urachus (arrow), highly suspicious for urachal malignancy.

In November 2025, the patient underwent partial cystectomy with en bloc urachal resection, umbilectomy, and bilateral pelvic lymph node dissection. Gross examination revealed a 6-cm tumor involving the bladder dome and urachal remnant. Histopathological evaluation demonstrated moderately differentiated urachal adenocarcinoma arising from the urachal remnant. The tumor invaded the bladder wall and extended into the perivesical soft tissue. Perineural invasion and tumor necrosis were identified, whereas lymphovascular invasion was absent. Surgical margins were negative for tumor involvement. Examination of the dissected pelvic lymph nodes revealed no metastatic disease (0/14 lymph nodes positive; 10 right-sided and 4 left-sided lymph nodes).

Despite apparently complete surgical resection, surveillance evaluation approximately 6 months later demonstrated local recurrence. The patient subsequently underwent transurethral resection of the bladder tumor (TURBT) in May 2026. Histopathologic assessment of the TURBT specimen revealed high-grade urothelial carcinoma. Immunohistochemical analysis demonstrated strong CK7 positivity, positive GATA3 expression, weak CK20 positivity, and negative CK5/6 staining, supporting the diagnosis of high-grade urothelial carcinoma. For evaluation of the extent of recurrent disease, whole-body ^18^F-FDG PET/CT was performed approximately 1 month later. Maximum-intensity-projection and fused PET/CT images demonstrated a large hypermetabolic lobulated mass involving the anterior bladder wall, measuring 58 × 40 mm with central necrotic components and a maximum standardized uptake value (SUVmax) of 6.9, consistent with recurrent malignancy (Fig. 2, 3). The lesion exhibited close proximity to the adjacent pelvic wall musculature and sigmoid colon.

**Fig. 2.**
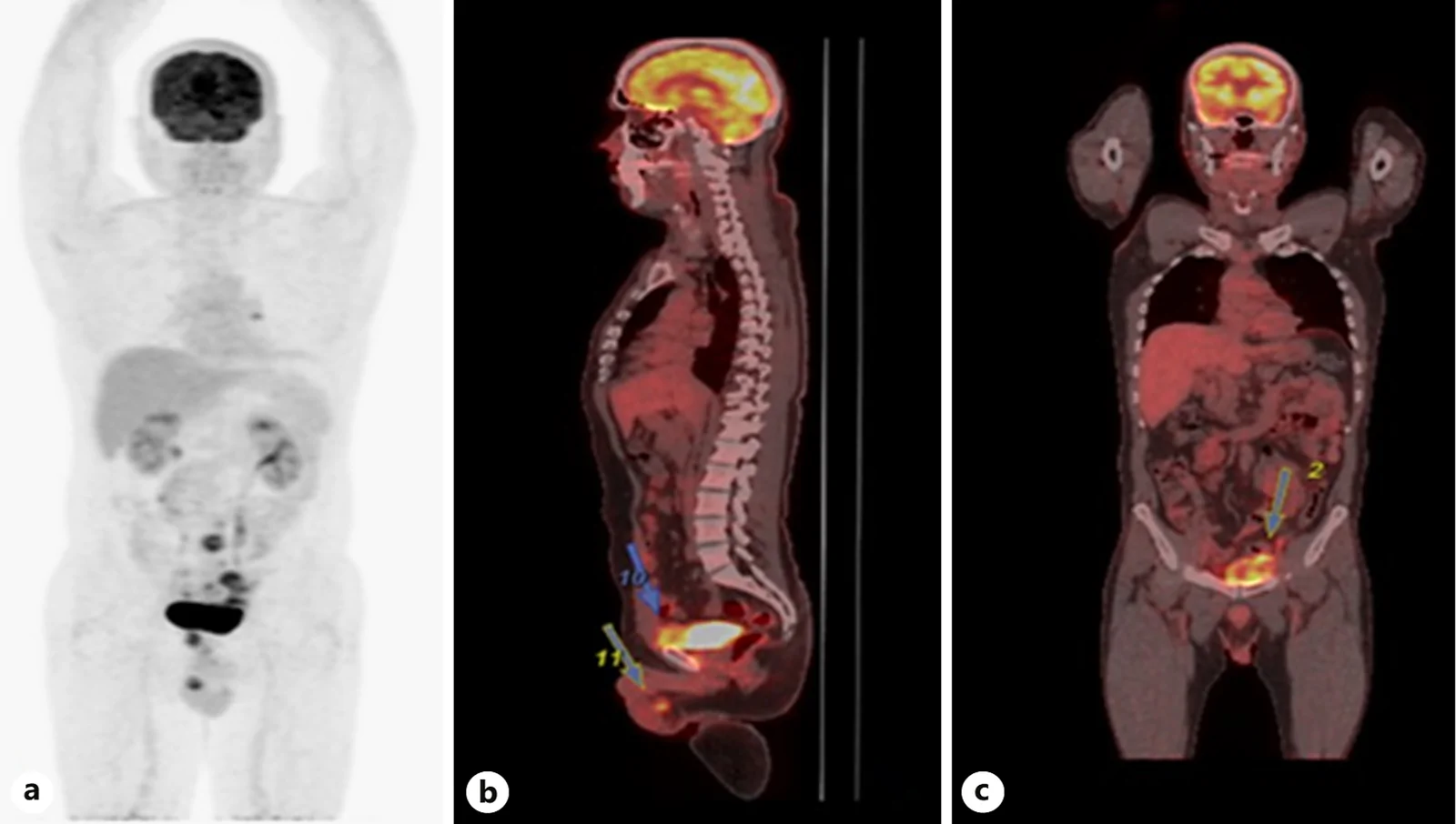
Whole-body ^18^F-FDG PET/CT findings at recurrence (SUVmax = 6.9) and suspicious right hemiscrotum (SUVmax = 6.4). **a** Maximum-intensity-projection (MIP) image demonstrating multifocal hypermetabolic lesions. **b** Sagittal fused PET/CT image showing the recurrent hypermetabolic bladder mass. **c** Coronal fused PET/CT image demonstrating the extent of recurrent pelvic disease and associated metastatic involvement.

**Fig. 3.**
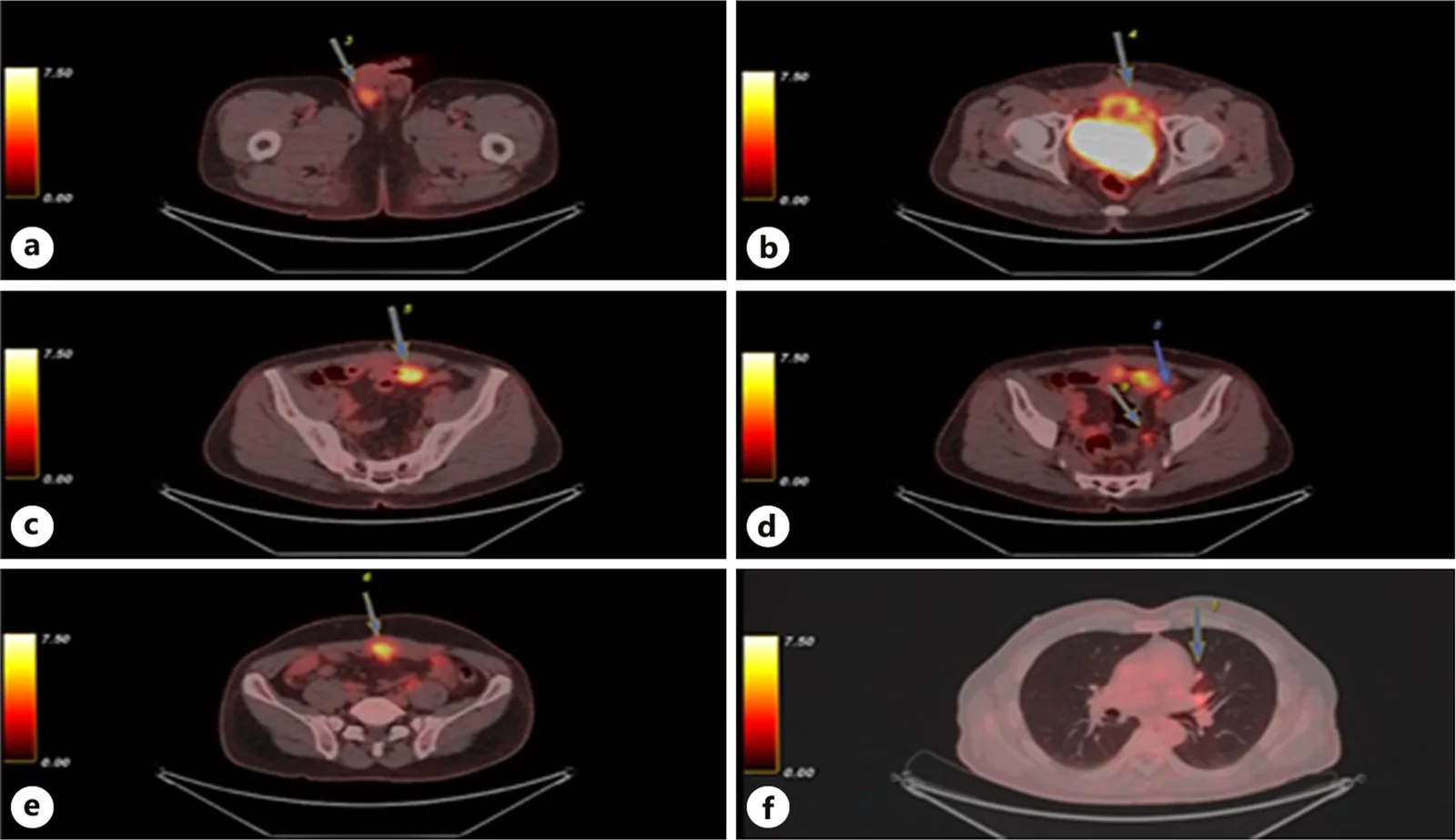
Axial fused ^18^F-FDG PET/CT images demonstrating sites of recurrent and metastatic disease. **a** FDG-avid soft-tissue lesion within the right hemiscrotum, suspicious for scrotal metastasis involvement (SUVmax = 6.4). **b** Recurrent hypermetabolic bladder mass (SUVmax = 6.9). **c** FDG-avid metastatic deposit within the pelvis (SUVmax = 6.1). **d** Hypermetabolic pelvic lymph node metastasis (SUVmax up to 2.9). **e** FDG-avid intraperitoneal metastatic deposit within the abdomen (SUVmax up to 3). **f** A 11-mm left upper lobe pulmonary nodule demonstrating mild FDG uptake (SUVmax 2.0), suspicious for metastatic involvement in the clinical context.

In addition to the primary recurrent lesion, PET/CT revealed multifocal metastatic disease. A hypermetabolic soft-tissue lesion was identified in the anterior midline abdomen at the level of the umbilicus, measuring approximately 20 mm with an SUVmax of 6.6. Additionally, multiple enlarged left common and external iliac lymph nodes with mild to moderate FDG uptake (SUVmax up to 2.9), multiple intraperitoneal metastatic deposits with mild to moderate FDG uptake (SUVmax up to 3), a hypermetabolic lesion within the right hemiscrotum demonstrating relatively high FDG uptake (SUVmax = 6.4) and an 11-mm left upper lobe pulmonary nodule with mild FDG uptake (SUVmax = 2.0). Although the pulmonary nodule demonstrated only mild FDG uptake, it was considered suspicious for metastatic involvement in the context of disseminated recurrent disease.

A particularly notable finding was a 15-mm hypermetabolic soft-tissue lesion within the right hemiscrotum demonstrating intense FDG uptake (SUVmax 6.4), highly suspicious for metastatic involvement (Fig. 3). A suspected metastatic scrotal lesion from urachal malignancy is exceedingly uncommon and represented one of the most distinctive features of the present case. Furthermore, thoracic imaging revealed an 11-mm pulmonary nodule in the left upper lobe with mild FDG uptake (SUVmax = 2.0). Although the degree of FDG uptake was relatively low, the nodule was considered suspicious for metastatic involvement in the context of widespread recurrent disease (Fig. 3). An additional 10-mm right lower lobe pulmonary nodule without significant FDG uptake was also identified.

Collectively, the PET/CT findings demonstrated extensive recurrent and metastatic disease involving the bladder, abdominopelvic cavity, pelvic soft tissues, lung, and scrotum. The imaging results substantially upstaged the disease burden and provided comprehensive characterization of the unusual metastatic distribution. Following multidisciplinary evaluation, the patient was referred for further oncologic management. The chronological sequence of the patient’s clinical course, including the initial diagnosis, surgical management, recurrence, pathological findings, and subsequent ^18^F-FDG PET/CT evaluation, is summarized in Table 1.

**Table 1.** Timeline of the patient’s clinical course from initial diagnosis to detection of recurrent metastatic disease

Date	Clinical event
November 2025	Contrast-enhanced CT demonstrated a urachal mass arising from a persistent urachal remnant at the bladder dome
November 2025	Partial cystectomy with en bloc urachal resection, umbilectomy, and bilateral pelvic lymph node dissection was performed
Postoperative pathology	Moderately differentiated urachal adenocarcinoma with perivesical extension and perineural invasion; surgical margins and all 14 pelvic lymph nodes were negative
Approximately 6 months later	Surveillance evaluation demonstrated local recurrence
May 2026	Transurethral resection of the bladder tumor (TURBT) was performed. Histopathology revealed high-grade urothelial carcinoma with supportive immunohistochemical findings
June 2026	Restaging 18F-FDG PET/CT demonstrated a recurrent bladder mass, pulmonary nodules suspicious for metastatic involvement, multiple intraperitoneal metastatic deposits, pelvic lymph-node and soft-tissue lesions, and a right hemiscrotal lesion highly suspicious for metastatic involvement
After multidisciplinary evaluation	The patient was referred for further oncologic management

Overall, the patient’s clinical course progressed from the initial diagnosis of urachal adenocarcinoma and surgical resection with curative intent to subsequent urinary bladder recurrence, which was histopathologically diagnosed as high-grade urothelial carcinoma. Restaging with ^18^F-FDG PET/CT then demonstrated recurrent pelvic disease, pulmonary nodules suspicious for metastatic involvement, peritoneal metastatic deposits, pelvic soft-tissue lesions, and a presumed right hemiscrotal metastatic lesion, indicating disseminated disease progression.

The authors have completed the CARE Checklist for this case report, which is available as online supplementary Material (for all online suppl. material, see https://doi.org/10.1159/cro/abzag038).

## Discussion

Urachal adenocarcinoma is a rare and biologically aggressive malignancy arising from persistent embryologic remnants of the urachus. Owing to its low incidence, most available evidence is derived from retrospective institutional series and isolated case reports rather than prospective studies [1, 3, 4]. Despite advances in surgical management, recurrence and metastatic dissemination remain major causes of treatment failure. The present case illustrates several clinically important aspects of this uncommon disease, including recurrence following apparently curative surgery, discordant histopathologic findings at recurrence, extensive metastatic dissemination, and an unusual FDG-avid scrotal lesion detected during restaging PET/CT.

Complete surgical excision with partial cystectomy and en bloc resection of the urachus and umbilicus is considered the standard treatment for localized urachal adenocarcinoma [1, 6]. Our patient underwent margin-negative resection with negative pelvic lymph nodes, findings generally associated with improved oncologic outcomes. Nevertheless, recurrent disease developed within approximately 6 months of surgery. Early recurrence despite apparently adequate surgical management has been reported in previous series and reflects the aggressive biological behavior of some urachal tumors [5, 6]. Several adverse prognostic factors have been associated with inferior outcomes, including advanced pathologic stage, nodal involvement, positive surgical margins, metastatic disease at presentation, and local extension beyond the bladder [5, 6]. Although lymphovascular invasion was absent in our patient, the presence of perineural invasion and extension into perivesical soft tissue may indicate a more aggressive tumor phenotype and may have contributed to the rapid recurrence observed in this case.

An additional noteworthy feature was the discrepancy between the histopathologic findings of the primary and recurrent tumors. The initial surgical specimen demonstrated moderately differentiated urachal adenocarcinoma, whereas the recurrent TURBT specimen showed high-grade urothelial carcinoma with immunohistochemical positivity for CK7 and GATA3. The biological significance of this finding remains uncertain. Several explanations may be considered, including intratumoral histologic heterogeneity, divergent differentiation within the recurrent neoplasm, sampling-related differences between specimens, or the development of a distinct urothelial malignancy in the residual bladder. Because comprehensive molecular characterization was not available, definitive conclusions regarding the relationship between these two pathologic entities cannot be established. Nevertheless, this observation highlights the diagnostic complexity that may occasionally be encountered during the follow-up of urachal malignancies and underscores the importance of repeat histopathologic evaluation in recurrent disease [4, 5].

The most remarkable aspect of the present case was the presence of an FDG-avid scrotal lesion in the setting of recurrent and metastatic urachal carcinoma. Urachal adenocarcinoma most commonly metastasizes to the lungs, liver, lymph nodes, bone, and peritoneum [3, 5, 7]. In our patient, pulmonary nodules and multiple intraperitoneal metastatic deposits were identified, consistent with previously described metastatic patterns. However, scrotal involvement is exceptionally uncommon. Although unusual metastatic sites have been described, including breast and ovarian metastases, involvement of scrotal structures appears to be extraordinarily rare [9, 10]. Selected reports of unusual metastatic patterns and relevant imaging findings in urachal and urothelial malignancies are summarized in Table 2.

**Table 2.** Selected reports of unusual metastatic patterns and relevant imaging findings in urachal and urothelial malignancies

Author (year)	Primary tumor	Metastatic site	Imaging modality	Key findings	Relevance to present case
Das et al. [7] (2021)	Urachal adenocarcinoma	Multiple metastatic sites	^18^F-FDG PET/CT	PET/CT identified additional lesions and altered staging	Supports the clinical value of FDG PET/CT in restaging urachal carcinoma
Stokkel et al. [8] (2021)	Urachal carcinoma	Recurrent/metastatic disease	^18^F-FDG PET/CT	PET/CT improved detection of metastatic disease	Demonstrates the diagnostic contribution of PET/CT beyond conventional imaging
Zhao et al. [10] (2016)	Urachal adenocarcinoma	Breast	CT, pathology	Metastatic urachal adenocarcinoma mimicking primary breast carcinoma	Demonstrates the ability of urachal adenocarcinoma to metastasize to highly unusual anatomical sites
Turo et al. [11] (2014)	Urothelial carcinoma of the bladder	Testis	Ultrasound, pathology	Extremely rare testicular metastasis from bladder cancer	Highlights the rarity of metastatic spread to scrotal contents
Fukagawa et al. [12] (2022)	Urothelial carcinoma of the bladder	Testis	CT, ultrasound, pathology	Delayed testicular metastasis after bladder cancer treatment	Supports the possibility of unusual genitourinary metastatic pathways
Liu et al. [13] (2024)	Urothelial carcinoma	Testis	Imaging and pathology	Literature review of testicular metastases from urothelial carcinoma	Provides contemporary evidence regarding rare scrotal/testicular involvement
Issa et al. [14] (1994)	Transitional cell carcinoma of the bladder	Spermatic cord	Surgical pathology	Metastatic involvement of the spermatic cord	Anatomically relevant to the present case of scrotal metastasis
Hirano et al. [15] (2015)	Various primary malignancies	Spermatic cord	CT, pathology	Review of metastatic spermatic cord tumors	Demonstrates the exceptional rarity of metastatic spread to scrotal structures

Several mechanisms may account for metastatic spread to the scrotal region. Hematogenous dissemination is a plausible route, particularly in patients with widespread systemic metastatic disease. Retrograde lymphatic spread through interconnected pelvic, iliac, inguinal, and scrotal lymphatic channels may also contribute, especially when pelvic lymph nodes are involved. A further possible mechanism is transperitoneal dissemination through a persistent or partially patent processus vaginalis, which may permit malignant cells or tumor deposits within the peritoneal cavity to reach the scrotal structures. Direct extension is less likely unless there is locally advanced disease involving adjacent pelvic or inguinal tissues. In the present patient, the coexistence of pulmonary nodules, iliac lymph-node involvement, multiple intraperitoneal metastatic deposits, pelvic soft-tissue lesions, and the right hemiscrotal lesion suggests that hematogenous, lymphatic, and transperitoneal routes may all be considered. The presence of peritoneal disease particularly supports the possibility of transperitoneal spread, although the exact route cannot be established without histopathological and anatomical confirmation.

Previously reported metastases involving the scrotum, testis, or spermatic cord from genitourinary malignancies have generally occurred in patients with advanced or recurrent disseminated disease [11–15]. In this respect, the present case is consistent with earlier reports, as the right hemiscrotal lesion was identified together with pelvic lymph node involvement, peritoneal deposits, pulmonary nodules, and other pelvic metastatic foci. However, the present case differs from many previously published cases in which scrotal or paratesticular involvement was diagnosed after the development of a palpable mass, scrotal symptoms, surgical exploration, or histopathological examination. In our patient, the lesion was detected during systemic restaging with ^18^F-FDG PET/CT and was interpreted as a presumed metastatic focus based on its FDG uptake, anatomical appearance, and the overall distribution of disease. Furthermore, the coexistence of recurrent urachal adenocarcinoma and a subsequent high-grade urothelial carcinoma adds diagnostic complexity not commonly emphasized in earlier reports. Because histopathological confirmation of the scrotal lesion was unavailable, direct comparison of tumor histology with previously reported cases remains limited.

Another important feature of this case is the contribution of ^18^F-FDG PET/CT to disease assessment. While CT remains the primary imaging modality for diagnosis and surveillance of urachal carcinoma, recent studies have demonstrated that FDG PET/CT may provide additional clinically relevant information, particularly in recurrent or metastatic disease [7, 16, 17]. In the present patient, PET/CT demonstrated the recurrent bladder mass and identified pulmonary nodules suspicious for metastatic involvement, multiple intraperitoneal deposits, pelvic lesions, and an unusual right hemiscrotal lesion suspicious for metastatic involvement. Multiple intraperitoneal metastatic deposits, pelvic metastatic foci, and the unusual FDG-avid scrotal lesion. These findings contributed to a more comprehensive assessment of disease extent and influenced staging and therapeutic planning. Our observations therefore support the growing evidence that FDG PET/CT may serve as a valuable adjunctive imaging modality in selected patients with suspected recurrent urachal carcinoma.

In the present case, the degree of FDG uptake varied among the different lesions. While the recurrent bladder mass and the right hemiscrotal lesion demonstrated relatively high FDG uptake, the iliac lymph nodes and peritoneal deposits showed mild to moderate FDG uptake, and the pulmonary nodule exhibited only mild FDG uptake. Therefore, interpretation of the PET/CT findings was based on the overall pattern of disease distribution, the corresponding anatomical findings on CT, and the clinical context of disseminated recurrent disease rather than SUVmax values alone.

Management of recurrent metastatic urachal carcinoma remains challenging because of the rarity of the disease and the absence of prospective randomized trials or a universally accepted standard systemic regimen [1, 3, 6]. Treatment is therefore individualized according to disease extent, resectability, prior therapy, molecular characteristics, and the patient’s performance status. Systemic chemotherapy commonly includes fluoropyrimidine- and platinum-containing regimens, such as combinations based on 5-fluorouracil, oxaliplatin, or cisplatin. Surgical resection or local treatment may be considered in carefully selected patients with isolated or oligometastatic, technically resectable disease, whereas disseminated recurrence is generally managed with systemic therapy and supportive care [1, 3, 6]. Molecular profiling may identify potentially actionable alterations or biomarkers for targeted therapy or immunotherapy, although evidence for these approaches remains limited [3]. Prognosis is strongly influenced by tumor stage, resectability, and metastatic burden [3, 5, 6]. Patients with recurrent metastatic disease generally have a poor prognosis, particularly in the presence of multisite dissemination, underscoring the importance of early detection, accurate staging, and multidisciplinary management.

## Conclusion

Urachal adenocarcinoma may recur aggressively despite apparently curative surgery and may occasionally demonstrate unusual patterns of metastatic dissemination. In the present patient, ^18^F-FDG PET/CT provided a comprehensive assessment of disease burden and identified a presumed right hemiscrotal metastatic focus, together with pulmonary nodules, intraperitoneal deposits, pelvic lymph nodes, and soft-tissue lesions suspicious for metastatic involvement. PET/CT contributed to disease staging and treatment planning but did not establish a definitive histopathological diagnosis. Histopathological examination remains the diagnostic gold standard whenever clinically feasible.

## Acknowledgment

The authors would like to thank the patient for providing written informed consent for publication of this case report.

## Statement of Ethics

Ethical approval was not required for this single-patient case report in accordance with the institutional policy of Tehran University of Medical Sciences. Written informed consent was obtained from the patient for publication of this case report and the accompanying images.

## Conflict of Interest Statement

The authors declare that they have no conflicts of interest.

## Funding Sources

This research received no specific grant from any funding agency in the public, commercial, or not-for-profit sectors.

## Author Contributions

Fatemeh Saboktakin: conceptualization, data collection, literature review, and writing of the original draft. Mohammad Reza Ghasri: PET/CT image interpretation, clinical data interpretation, and critical revision of the manuscript. Shaghayegh Nourbakhsh and Najmeh Ranjbar: data acquisition, literature review, and manuscript editing. Sarvenaz Mirzakhani: study supervision, case conceptualization, PET/CT image interpretation, critical revision of the manuscript for important intellectual content, and final approval of the version to be published. All authors read and approved the final manuscript.

## Data Availability

The data supporting the findings of this study are not publicly available because they contain information that could compromise patient privacy but are available from the corresponding author upon reasonable request.
